# First attempt success rate of intraosseous access in preterm infants and neonates: a systematic review

**DOI:** 10.1016/j.resplu.2026.101334

**Published:** 2026-04-16

**Authors:** Markus Ortner, Alexandra Kaider, Georg Heinze, Tamara Odar, Ewald Unger, Gunpreet Coudert Oberoi

**Affiliations:** a Centre for Medical Physics and Biomedical Engineering, Medical University of Vienna, Währinger Gürtel 18-20, 1090 Vienna, Austria; b SVAN Technologies GmbH, Herrengasse 25, 2700 Wiener Neustadt, Austria; c Institute of Clinical Biometrics, Centre for Medical Data Science, Medical University of Vienna, Vienna, Austria; d ADAX d.o.o., Bravničarjeva ulica 13, Ljubljana, Slovenia

**Keywords:** Infusions, Intraosseous, Infant, Newborn, Emergency treatment

## Abstract

**Objectives:**

While intraosseous (IO) access is an established and safe alternative route for vascular access in children and adults when intravenous (IV) access is not possible, its use in preterm and term neonates ≤28 days and young infants remains challenging. Success rates are lower in preterm infants and neonates due to anatomical and technical factors. We conducted a systematic literature review to examine the first-attempt success rates of tibial IO access in preterm and term neonates and young infants, comparing clinical outcomes with anatomical evidence to identify limitations, complications, and gaps in device suitability for this vulnerable population.

**Data sources:**

Data were obtained through systematic searches of PubMed, Ovid MEDLINE and the Cochrane Library, covering English-language studies. The search was conducted in December 2025, using predefined search terms.

**Study selection:**

Eligible studies included prospective or retrospective clinical and cadaveric studies and reported per‑leg first‑attempt outcomes involving preterm and term neonates and young infants ≤6 months and ≤8 kg, reporting success rates for drill-assisted tibial IO access. Manual-assisted access methods, simulation and animal studies were excluded.

**Data extraction:**

Two reviewers independently screened and extracted data on study design, population, device type, success rates, and complications.

**Data synthesis:**

A total of six studies were included, comprising 108 documented tibial intraosseous (IO) insertions. Clinical data accounted for 40 attempts, with 23 successful first attempts, while cadaveric data included 68 tibial sites, with 30 successful insertions. Using random-effects meta-analysis models applied separately to each study group, the combined first-attempt success rate in clinical studies was 54.1% [95% CI: 30.6–75.9%]. In cadaveric studies, the combined success rate was 44.1% [95% CI: 32.9–56.1%]. These success rates are substantially lower than those reported for older children and adults, in whom first-attempt IO placement typically exceeds 80–95% with drill-assisted systems. The decrease in preterm and term neonatal success reflects the anatomical, technical, and procedural challenges unique to this population.

**Conclusions:**

First-attempt intraosseous access success in neonates and young infants appears lower than in older paediatric populations, likely due to anatomical limitations and the use of devices not specifically designed for this patient group. Neonatal-specific device development and further prospective research are needed to improve procedural success and safety.

## Highlights

• **Question:** IO first attempt success rate in preterm/term neonates and device reliability?
• **Finding:** 1st attempt success 54.1% clinical, 44.1% cadaveric; lower in VLBW infants**.**
• **Meaning:** Neonatal-specific IO devices and structured training are urgently needed.

## Introduction

In paediatric emergencies, establishing rapid and reliable vascular access is a critical, yet often challenging step for administering life-saving fluids and medications.^1^, ^2^ The physiological stress of a serious medical condition frequently leads to peripheral vasoconstriction, making conventional intravenous (IV) cannulation difficult or impossible, particularly in infants and young children.^1^ The need for advanced resuscitation requiring vascular access is relatively rare event, occurring in approximately 0.05–0.12% of all births.^2^ The established standard of care, as outlined by the American Heart Association and the American Academy of Paediatrics in the 2025 Guidelines for Cardiopulmonary Resuscitation and Emergency Cardiovascular Care, prioritises peripheral IV cannulation. For neonatal resuscitation immediately after birth or up to one day after birth, the umbilical venous catheter (UVC) is the preferred primary route. If UVC attempts are unsuccessful or are likely to cause significant delay, intraosseous (IO) access is recommended as the alternative.^3^, ^4^, ^5^

Intraosseous (IO) access offers clinical benefits by providing rapid, reliable vascular access when intravenous attempts fail or are not possible, enabling fast administration of life-saving drugs and fluids.^3^, ^6^ Mileder et al. (2020) reported that while minor short-term complications, such as local reactions or extravasation, may occur, no severe or lasting adverse effects were observed, although the overall evidence base remains limited.^2^ A variety of intraosseous (IO) access devices are currently used in neonatal and paediatric emergency care, including manual options such as the Cook Needle (Cook Medical, USA), Butterfly IV needles or needles for biopsy or spinal/biopsy needles (e.g., Jamshidi®, Becton Dickinson, USA) as well as semiautomatic and powered systems like the Bone Injection Gun (PerSys Medical, USA) and Arrow EZ-IO (Teleflex, USA). Most currently available IO devices were originally developed for older children or adults and are used off-label in neonates weighing <3 kg. In particular, the Butterfly and spinal or biopsy needles are not intended for IO access and require highly skilled clinicians to insert them manually with precise control.^7^ Moreover, no IO device currently on the market is approved or specifically designed for infants weighing under 3 kg.^3^ Existing systems lack safety features necessary to prevent overpenetration and soft-tissue injury. These limitations may indicate a clinical need to develop and evaluate neonatal-specific IO access devices to ensure safer, more reliable vascular access in the smallest and most vulnerable patients.

## Methods

The review was conducted in accordance with PRISMA (Preferred Reporting Items for Systematic Reviews and Meta-Analyses) guidelines and adhered to predefined eligibility, search, and analysis criteria. The review was preregistered with PROSPERO (https://www.crd.york.ac.uk/PROSPERO/view/CRD420251208158) and additional methodological details are provided in the Annex.

### Inclusion and exclusion criteria

Eligible studies included those that reported data on preterm infants (≤37 weeks’ gestational age up to ≤28 days after birth) and/or term neonates or young infants up to 6 months of age with a body weight below 8 kg. Both live and cadaveric investigations were considered, provided that the proximal tibia was used as the insertion site for intraosseous (IO) access. Prospective and retrospective clinical or anatomical studies were included if they explicitly reported first-attempt success rates for drill-assisted IO access devices. Cadaveric studies were analysed separately from live clinical data.

Studies using manikins, simulation models, or animal data or models were excluded.

### Outcome of interest

The primary outcomes of interest included the first-attempt success rate for intraosseous (IO) access. Complication rates were evaluated, distinguishing between minor and severe events. Data were analysed by patient age group, with a focus on term and preterm neonates and infants, and subgroup outcomes were reported when available. Because clinical success was defined functionally, whereas cadaveric success required imaging-confirmed tip location, we report these data separately. Only drill-assisted attempts were included; manually performed attempts were not considered in the analysis.

### Definitions

The first-attempt success rate was defined as the successful placement of the intraosseous (IO) needle tip within the medullary cavity of the bone. The success rate was evaluated both on a per-patient basis and on a per-insertion-site basis. Per-patient first-attempt success refers to successfully establishing a functional line on the first attempt for each patient. Per-site or per-leg success refers to the success of each needle insertion, meaning one patient may have several recorded attempts if multiple insertions were required.

Complications were classified as either severe or minor. *Severe complications* are events that cause substantial tissue damage, necessitate major intervention, and may lead to long-term harm or limb loss, such as compartment syndrome, fracture, or osteomyelitis. *Minor complications* are localised issues like fluid leakage (extravasation), pain, or skin reactions that are generally self-limiting and resolve with minimal or no intervention, without causing lasting damage.

### Search strategy

A systematic search was conducted in December 2025 across several databases, including PubMed, Ovid MEDLINE, and the Cochrane Library, using predefined search terms related to intraosseous (IO) access, neonates, infants, and success rates. The search was limited to peer-reviewed publications in English from the past ten years to ensure the evidence reflects contemporary clinical practice. The search strategy adhered to PRISMA guidelines and focused on studies that met predefined inclusion and exclusion criteria.

### Data extraction

Two independent reviewers screened titles, abstracts, and full texts of identified studies. Data extraction was performed using a standardised template to ensure consistency. Extracted variables included study design, population characteristics, device type, insertion site, success rate (first and second attempt), and reported complications. Discrepancies between reviewers were resolved by discussion and consensus to maintain objectivity and accuracy in data handling.

### Quality assessment

The methodological quality of the included studies was evaluated using established frameworks. Clinical observational studies were assessed using the ROBINS-I tool to examine potential biases related to confounding, participant selection, intervention classification, missing data, outcome measurement, and reporting. Study selection was performed independently by two reviewers who screened titles, abstracts, and full texts against predefined inclusion criteria, with disagreements resolved by discussion. Cadaveric studies were included but analysed separately from clinical studies. Certainty of evidence was considered using GRADE-style assessments. All evaluations were conducted using Anara software (https://anara.com) to support a structured and transparent appraisal, and the results were independently reviewed and verified by the authors. Additional methodological details and supporting information are provided in the Appendix A.

### Statistical analysis

Data were summarised descriptively, and outcomes were standardised on a per-leg basis to enable comparison across studies. First-attempt success rates were calculated as the proportion of successful insertions relative to total attempts. Clopper-Pearson confidence intervals (CI) were calculated for the individual studies’ success rates. Combining of results was performed using a random-effects meta-analytic model. A random-effects model was selected to account for the expected between-study variability arising from differences in populations, operators, and study settings. Forest plots were generated to present the results of the meta-analyses.

## Results

**Fig. 1:**
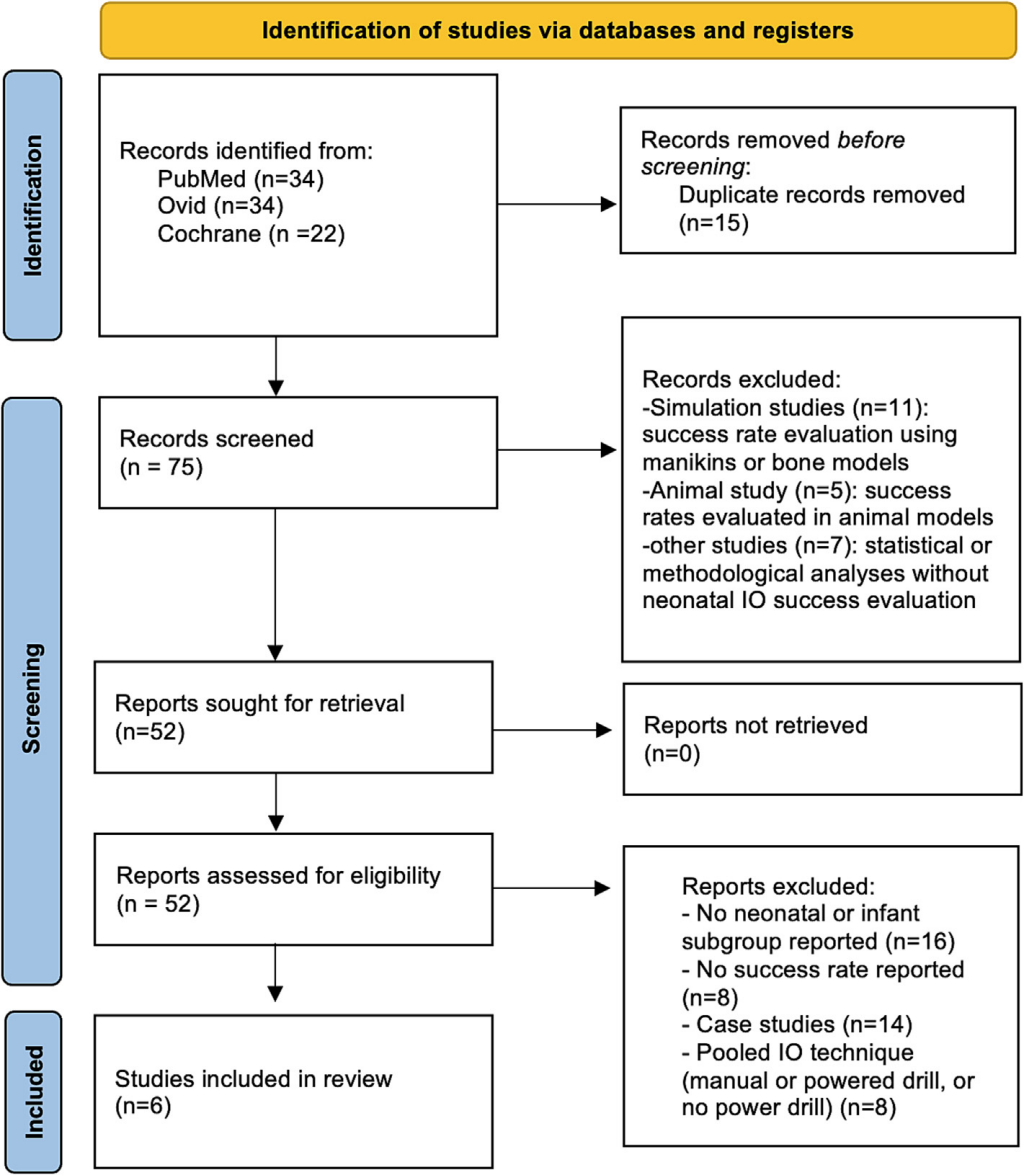
**Preferred Reporting Items for Systematic Reviews and Meta-Analyses (PRISMA) 2020 flow diagram for new systematic reviews, which included searches of databases and registers only**.

The systematic literature search was conducted and documented in accordance with the PRISMA guidelines. A total of 90 records were identified across PubMed, Ovid MEDLINE, and the Cochrane Library. After removing 14 duplicates, 76 unique studies were screened. Twenty were excluded based on the title and abstract as simulations, animal studies, or unrelated to the topic. Fifty-six full-text reports were retrieved for detailed evaluation, of which 50 were excluded because they lacked neonatal or infant subgroup data or presented paediatric outcomes. Finally, six studies met all inclusion criteria and were included in the systematic review, comprising both clinical (live) and cadaveric investigations (Fig. 1).

Several studies were excluded to maintain methodological rigour and ensure relevance to clinical practice. Simulation-based studies and animal models were excluded due to limited external validity. Studies using manual IO devices or other devices were also excluded to maintain consistency in device evaluation. Similar review articles like Scrivens et al. (2019) were excluded from the analysis to prevent data duplication, although they were consulted for completeness. Additionally, studies that included only infants under 1 year of age, without precise subgroup analyses for neonates (≤28 days) or preterm infants (≤37 weeks), were excluded. Studies, which reported pooled data for infants but did not separate the outcomes specifically for the neonatal subgroup were also excluded.^8^, ^9^, ^10^

### Summary of studies regarding per-patient or per-leg

The following tables distinguish between cadaveric and clinical studies that evaluate the success of intraosseous (IO) access in neonatal and paediatric populations.

Studies included in this review report intraosseous (IO) access success using two primary metrics: per-patient and per-leg.

• Per-leg means that each tibial insertion (one per tibia) is treated as an independent attempt. This approach allows assessment of success for each leg, typically corresponding to a single insertion attempt per tibia.
• Per-patient means that results are summarised at the level of each patient, even if multiple insertion attempts were made on the same or opposite tibia. In these cases, overall success is defined as whether IO access was ultimately achieved for that patient, regardless of the number of attempts (Table 1, Table 2, Table 3).

**Table 1:** Cadaveric studies evaluating intraosseous (IO) access accuracy in neonatal populations.

**Study authors**	**Study model**	**Patient/subject population (P)**	**Success metric**
**Cadaveric**			
Sengasai et al. (2024)	Prospective evaluation on neonatal cadavers using CT scans	*P* = 19 Asian neonatal cadavers, providing 38 tibiae for analysis. 19 tibias with EZ-IO and the other 19 via manual insertion with Acufirm sternal puncture needle. For Birthweight under 1500 g; 12 tibias in total, 6 EZIO and 6 Acufirm (manual)	**Per-Leg:** Success was confirmed by CT scan, requiring the needle tip to be in the marrow cavity and observing the distribution of contrast media
Harcke et al. (2020)	Retrospective review using Postmortem Computed Tomography (PMCT)	*P* = 30 deceased paediatric subjects under 6 months	**Per-leg:** Success was defined as the needle tip being located within the medullary portion of the bone, as visualised on PMCT scans
Fuchs et al. (2018)	Anatomical investigation on stillborn using spectral CT scans	*P* = 16 formaldehyde-fixed stillborn (median gestational age 29.2 weeks). Insertions were performed on both legs of each subject for EZ-IO drilled	**Per-Leg:** Success was defined as the exclusive position of the needle within the bone marrow, confirmed by contrast medium distribution on CT scans

**Table 2:** Studies from clinical data: evaluating intraosseous access (IO) success in neonatal patients.

**Study author(s)**	**Study model**	**Patient/subject population**	**Success metric**
**Clinical**			
Pifko et al. (2018)	Retrospective observational review in a Paediatric Emergency Department (PED)	*P* = 50 live paediatric patients, resulting in 77 total insertion attempts. Subjects were stratified by weight (≤8 kg vs. >8 kg)	**Per-patient:** Success was defined as the documented infusion of medications or fluids. Rates were calculated for both first attempts and total attempts
Mileder et al. (2020)	Questionnaire survey combined with a retrospective chart review in a single NICU	*P* = 12 live neonates who received 15 insertion attempts	**Per-patient:** The primary success rate was based on the number of patients (9 out of 12) who received successful access, defined as correct puncture and successful administration of fluid/medication
Rijnhout et al. (2024)	Single-centre retrospective cohort study at a Level 1 trauma centre	*P* = 188 live patients (73 children and 115 adults) with 232 total insertions analysed	**Per-patient:** Success was defined as documented aspiration and smooth infusion, or a clinically sufficient position for infusion. The rate was based on all 232 needle placements

**Table 3:** First-attempt success rates for intraosseous (IO) access in neonatal and paediatric populations, normalised per tibial site (per-leg basis). Calculation of success rate based on meta-analysis. Only success rates of drill-assisted devices were included; manual drills were excluded.

**Study**	**Type of study**	**Population**	**Total Attempts per tibial site**	**Number of first attempt success**	**First attempt success rate**
**Cadaveric**					
Sengasai et al. (2024)	Cadaveric	Birthweight under 1500 g	6	3	50% [95% CI: 11.81–88.19%]
Harcke et al. (2020)	Cadaveric	≤6 month	30	14	46.67% [95% CI: 28.34–65.67%]
Fuchs et al. (2018)	Cadaveric	Median Gestational Age (GA) 29.2	32	13	40.62% [95% CI: 23.70–59.36%]

**Clinical**					
Pifko et al. (2018)	Clinical	Infants ≤8 kg	7	2	28.57% [95% CI: 3.67–70.96%]
Mileder et al. (2020)	Clinical	<1 month	12	6	50% [95% CI: 21.09–78.91%]
Rijnhout et al. (2024)	Clinical	<0.5 years	21	5	71.43% [95% CI: 47.82–88.72%]

To ensure comparability across studies, we standardised all reported outcomes to a per-leg basis and calculated the Clopper-Pearson confidence intervals (CI). This standardisation was achieved by including only first-attempt success rates, excluding combined or overall success measures, to ensure all results correspond specifically to single per-leg insertion attempts.

### Success rate calculation

**Fig. 2:**
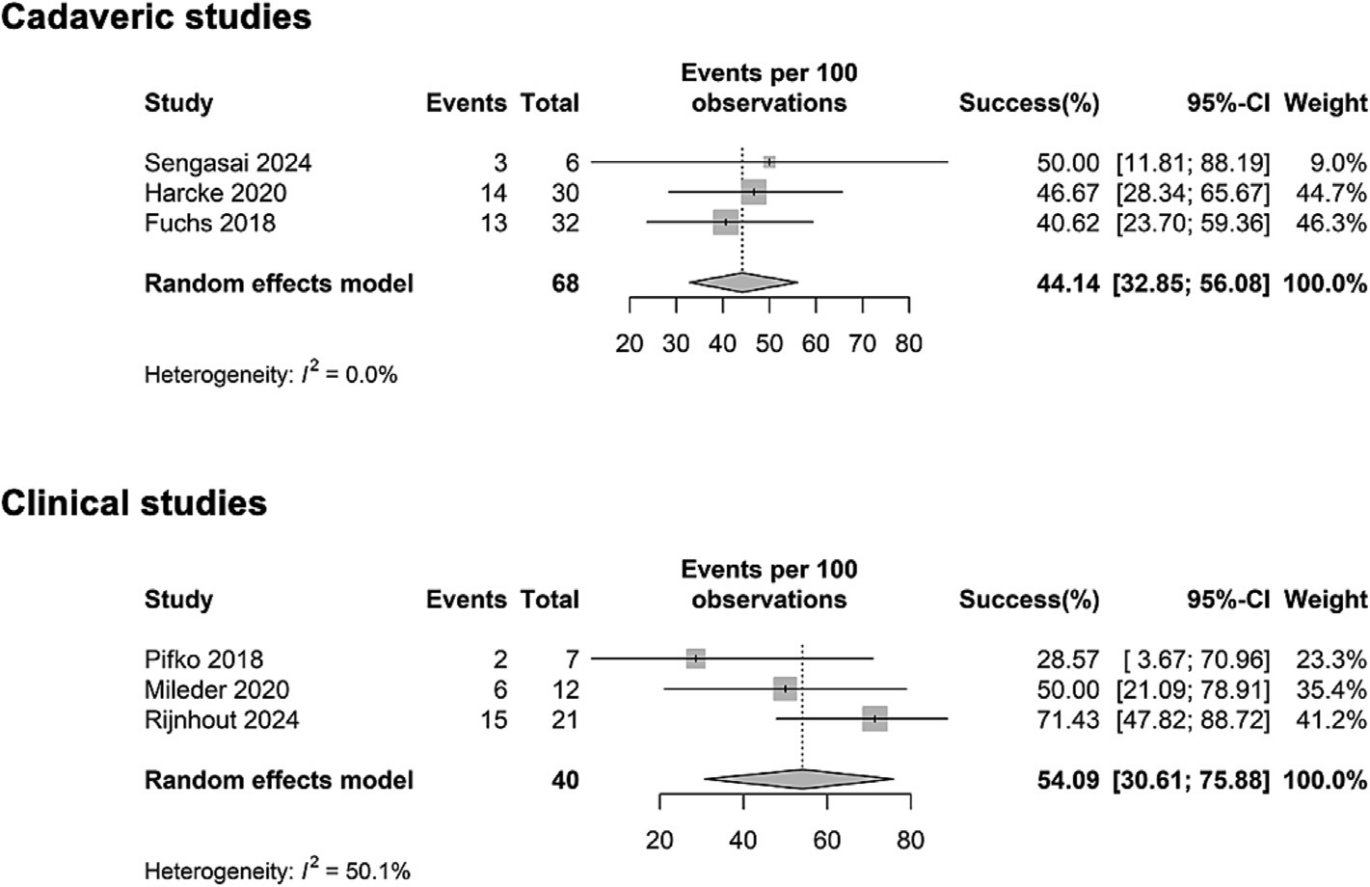
**Forest plots summarising the effect estimates from included studies. The first forest plot presents results from cadaveric studies, the second from clinical studies**.

Using random effects meta-analysis models, we calculated the combined success rate separately for each study group, cadaveric and clinical, based on the reported first-attempt outcomes for every individual study (Fig. 2).

Across six included studies, a total of 40 IO attempts per leg and 68 cadaveric tibial sites were evaluated. In live neonates and infants, the combined first-attempt success rate was 54.1% [95% CI: 30.6–75.9%]. In cadaveric studies, the combined success rate was 44.1% [95% CI: 32.9–56.1%]. A moderate heterogeneity (*I*^2^ = 50.1%) was observed in the clinical combined analysis, reflecting the wide variation in patient age groups, IO device types, and operator experience across the included studies.

### Complications

#### Cadaveric studies

Sengasai et al. found overall contrast leakage in 15.8% of tibial insertions, increasing to 41.7% in very-low-birthweight infants and occurring more often with the drill-assisted device than with the manual needle, although not statistically significant; no fractures were observed. Fuchs et al. and Harcke et al. reported no complications in their study.

#### Clinical studies

Mileder et al. reported minor short-term complications in 33.3% of successful neonatal IOs (paravasation, local skin reactions, soft-tissue infection) and no severe complications. Rijnhout et al. reported an overall complication rate of 7.7% across 232 attempts. Most were minor: extravasation occurred in 1.3%, and 1.7% IO catheters were removed due to pain after placement. More serious issues were rare: two patients (0.86%) were diagnosed with compartment syndrome after IO insertion (though not judged directly related to the puncture itself). There was one case (0.43%) in which the IO may have penetrated the growth plate. In Pifko et al., among 50 paediatric patients with at least one IO attempt, two extravasations (4%) after EZ-IO placement were reported.

## Discussion

This systematic review is, to our knowledge, the first to quantify first‑attempt tibial intraosseous (IO) success specifically in preterm and term neonates and very young infants using drill‑assisted systems, while separating clinical from cadaveric evidence. Across six included studies (108 tibial attempts), clinical (live) first‑attempt success was 54.1% and cadaveric first‑attempt success was 44.1%. These rates are far below the 80–94% first‑attempt success rates frequently reported in older children using drill‑assisted devices, underscoring unique neonatal challenges in landmarking, needle selection, and depth control.^8^, ^10^, ^11^

Both the AHA 2025 neonatal life support guidelines and the ERC 2025 newborn life support guidelines reaffirm umbilical venous catheterisation (UVC) as the preferred intravascular route at birth, with IO recommended when UVC cannot be established rapidly, particularly outside the immediate delivery room window or when expertise/equipment are limited.^5^ Neither body identifies neonatal‑specific IO devices as standard, and both highlight the evidence gap for direct UVC vs IO comparisons in human neonates.

Importantly, our clinical estimate sits lower than the largest neonatal clinical series to date: a German nationwide surveillance study of 161 neonates reported 91% overall success and 75% first‑attempt success (predominantly proximal tibia, mostly powered devices).^6^ A complementary 14‑year neonatal and paediatric retrieval cohort from New South Wales reported 102 neonates with a median post‑menstrual age of 39 ± 3 weeks and an overall IO procedural success of 87.8% during inter‑facility transport,^12^ again showing that, in well‑trained systems with ready device availability, IO access can be established rapidly and reliably even in very young infants. Differences between our estimate and this registry likely reflect (i) stricter first-attempt/per-leg normalisation in our review, (ii) our focus on clearly defined neonatal and low-weight infant subgroups rather than aggregated paediatric populations and (iii) restriction of the analysis to drill-assisted IO devices without combining outcomes across different device types. Nevertheless, the registry demonstrates that live neonatal IO success can approach approximately 75% on the first attempt in real-world systems with adequate training and device availability.

A recent single‑centre, retrospective cohort study, spanning adults and children, similarly cautioned that IO success is lower in infants <6 months, aligning with our observation that the youngest patients fare worse than older infants/children, likely due to bone size, cortical thickness, and limited medullary width.^3^

Cadaveric CT studies provide a mechanistic explanation for failures and complications. In Asian neonatal cadavers, overall placement success reached 86.8% but fell to 66.7% in very‑low‑birth‑weight (VLBW) infants; contrast leakage was common in the smallest bodies and more frequent with certain tips (e.g., EZ‑IO) that may overpenetrate beyond the narrow medullary cavity. These data quantify the tight margin for error in preterm/VLBW tibiae and the inadequacy of adult‑designed tips at neonatal scales.^13^

Beyond device tip geometry, the insertion landmark also matters. An ultrasound pilot in term and preterm newborns showed that “standard paediatric” distances (e.g., 10 mm distal to the tibial tuberosity) often violate an assumed 10 mm safety distance from the growth plate, suggesting that a slightly more distal/medial target (e.g., in 3–4 kg neonates, a median 13 mm distal and 6 mm medial) better balances access to the widest diaphyseal diameter with physeal safety.^14^ These data also document how tibial dimensions scale with weight, supporting weight‑adapted insertion depths.

Collectively, the anatomical evidence explains our live‑patient results: thin cortices, shallow skin‑to‑marrow distances, and very narrow medullary cavities increase the likelihood of overpenetration, malposition, and extravasation when using off‑label, adult‑derived systems with fixed needle lengths and aggressive bevels.

Within the studies included in this review, complications were infrequently reported and predominantly minor. Mileder et al. described minor short-term complications in 33.3% of successful neonatal IO insertions, such as paravasation, local skin reactions, and soft-tissue infection, with no severe events observed. Rijnhout et al. reported an overall complication rate of 7.7%, with extravasation in 1.3% of attempts and two cases of compartment syndrome (0.86%), though neither was judged to be directly caused by the IO puncture itself. One insertion was suspected to have reached the growth plate (0.43%). These findings suggest that, within the limited evidence from included studies, the procedural risk profile in neonates is characterised primarily by minor local complications, with severe events rare but not absent.

The broader published literature, beyond the studies included in this review, provides additional context. The Schwindt et al. nationwide German surveillance study reported potentially severe complications in approximately 6% of neonatal IO cases, including extravasation, necrosis, and fracture.^6^ In a large cross-sectional survey of Scandinavian emergency providers, extravasation was reported in 3.7% and compartment syndrome in 0.6% of IO cases across all age groups.^15^ It should be noted that these data are drawn from broader paediatric and adult populations and were not part of the systematic analysis in this review. Infectious complications such as osteomyelitis, growth plate injury, and fat embolism have also been described in the wider literature, though their incidence in neonates specifically is not well characterised.^16^

Case reports have documented rare but severe outcomes, including limb ischemia and loss, following IO placement in infants, attributed to malposition, posterior cortical penetration, or dislodgement under high-pressure infusion.^16^, ^17^ These events, while serious, represent exceptional outliers in the published record rather than a systematic risk quantified by the included studies. Their inclusion here is intended to illustrate the upper bound of potential harm, not to characterise the typical complication profile. The risk of severe complications appears highest in the smallest infants and in the context of prolonged or high-pressure infusion through a mispositioned needle.

Current mechanical devices (EZ‑IO, BIG (bone injection gun), FAST1™ intraosseous infusion system and NIO (New intraosseous)^18^) primarily mitigate this risk through operator technique, depth markings or preset depth heuristics, and manual monitoring, not through real-time sensing. Mitigation strategies informed by our synthesis and external data include: (i prioritise UVC during the delivery‑room phase where teams/equipment permit; (ii) use IO early when UVC delays are likely (e.g., outside the delivery room, limited expertise) as recommended by AHA/ERC 2025; (iii) select thinner and shorter needles and consider patient age specific drills or low‑torque insertion in ELBW/VLBW; (iv) target anatomically validated sites and weight‑adapt depth; (v) ensure easy flush/infusion, and continuously reassess the limb; (vi) train neonatal teams regularly and stock appropriately sized IO kits. This supports the rationale for neonatal‑specific IO designs incorporating smart real-time sensing and safety features to reduce over penetration and operator‑dependent failure modes.

For preterm and term neonates at birth, the first‑line remains UVC, with IO as the rescue route if UVC is not feasible swiftly. For neonates/infants beyond the immediate delivery room, IO becomes a pragmatic primary option when IV fails or is expected to be delayed, provided teams are trained and devices are available. Clinicians should temper expectations of first‑attempt success in preterm/VLBW infants and proactively implement the mitigation bundle above.

### Limitation

Only a few studies met the inclusion criteria, leading to limited evidence. Moreover, the included studies varied in design, comprising both clinical and cadaveric research, and employed different definitions of procedural success. Data on neonatal subgroups were often scarce within larger paediatric groups. Lastly, most procedures involved devices originally intended for older children or adults and used off-label in neonates, which may have affected the reported success rates.

Furthermore, the certainty of evidence is limited. Risk of bias assessment using the ROBINS-I framework for clinical observational studies indicated a moderate to serious risk of bias, mainly due to retrospective design, incomplete documentation, and potential confounding factors. Cadaveric studies provided high accuracy through imaging confirmation but are limited in their translation to clinical practice. Overall, the GRADE-style certainty assessment indicated low to moderate certainty for anatomical findings and low certainty for clinical outcomes, primarily due to small sample sizes and heterogeneous study designs. Detailed assessments are provided in the Appendix A.

## Conclusion

First-attempt tibial intraosseous access success in preterm and term neonates and young infants appears significantly lower than reported for older paediatric populations. These findings likely reflect anatomical constraints and the use of devices not specifically designed for neonatal patients. While intraosseous access remains an important emergency access method, when other vascular access routes fail, the results emphasise the need for neonatal-specific device designs and further prospective studies to improve procedural success and safety.

## Ethics statement

Ethical approval was not required for this systematic review of published literature and cadaveric/anatomical studies previously approved by their originating institutions (where applicable).

## Registration

Systematic review protocol registered on PROSPERO: CRD420251208158. (https://www.crd.york.ac.uk/PROSPERO/view/CRD420251208158).

## Patents

The device under development builds on the patent application WO 2024/100513 A1 (PCT/IB2023/061156), Apparatus for penetrating through an anatomical structure, for which Ewald Unger is listed as a co‑inventor; this application is related to penetration/IO‑type instrumentation (see patent document provided). The patent is owned by the Medical University of Vienna.

## Disclaimer

The views expressed are those of the authors and do not necessarily reflect those of the FFG. The sponsor/funder had no influence on the content beyond financial support as detailed above.

## Declaration of generative AI and AI-assisted technologies in the manuscript preparation process

During the preparation of this work, the authors used https://app.grammarly.com/ to improve grammar, spelling, and clarity in English, as well as anara.com to assist with literature navigation and organisation. After using these tools/services, the authors reviewed and edited the content as needed and take full responsibility for the content of the published article.

## Role of the sponsor/company involvement

The review was undertaken to inform benchmark selection for the control group in forthcoming clinical and pre-clinical investigations related to the development of neonatal intraosseous access devices (SVAN). To mitigate potential bias, the review followed a pre‑specified protocol and dual independent screening/extraction with independent statistical oversight from the Institute of Clinical Biometrics.

## CRediT authorship contribution statement

**Markus Ortner:** Writing – review & editing, Writing – original draft, Visualization, Data curation, Conceptualization. **Alexandra Kaider:** Writing – review & editing, Visualization, Formal analysis, Data curation. **Georg Heinze:** Writing – review & editing, Formal analysis, Data curation. **Tamara Odar:** Writing – review & editing, Validation, Supervision. **Ewald Unger:** Writing – review & editing, Conceptualization. **Gunpreet Coudert Oberoi:** Writing – review & editing, Writing – original draft, Visualization, Supervision, Funding acquisition, Formal analysis, Conceptualization.

## Funding

This work was supported by the 10.13039/501100004955Austrian Research Promotion Agency (FFG), Spin‑off Fellowship Program [FFG No. 909600]. The funder had no role in study design; data collection, analysis, or interpretation; manuscript preparation; or the decision to submit for publication. The corresponding author had full access to all the data and had final responsibility for the decision to submit.

## Declaration of competing interest

Gunpreet Coudert Oberoi (G.C.O.) is Chief Executive Officer and a shareholder of SVAN Technologies GmbH (Vienna, Austria). Ewald Unger (E.U.) is a shareholder of SVAN Technologies GmbH. Markus Ortner (M.O.) and G.C.O. are employees of SVAN Technologies GmbH and hold part‑time appointments at the Medical University of Vienna.

SVAN Technologies GmbH is developing a neonatal intraosseous access device; this systematic review was undertaken to define a benchmark control rate for planned pre-clinical and clinical investigations. No SVAN device was evaluated in this review, and no proprietary or non‑public data were used.

Tamara Odar is employed by ADAX d.o.o., Slovenia. ADAX d.o.o. acted as a contract research organisation (CRO) for this study under contract with the company SVAN.

Alexandra Kaider and Georg Heinze are employed at the Institute of Clinical Biometrics, Centre for Medical Data Science, Medical University of Vienna, Vienna, Austria, and contributed to this work in their roles as statisticians. They have no involvement with, nor do they have any financial or non-financial interests in, the company SVAN.

## Data Availability

The full search strategies, screening forms, data‑extraction templates, and the aggregated dataset used for synthesis are available from the corresponding author upon reasonable request.

## Appendix A Supplementary material

Supplementary Data 1 PRISMA 2020 checklist, search strategies, selection and data collection procedures, and risk-of-bias and GRADE-style certainity assessments for included studies.
