# Supplementary material for: First attempt success rate of intraosseous access in preterm infants and neonates: a systematic review
Source: Resusc Plus. 2026 Apr 16;29:101334. doi: 10.1016/j.resplu.2026.101334 (PMC13138175; doi:10.1016/j.resplu.2026.101334)
Supplement: Supplementary Data 1 — PRISMA 2020 checklist, search strategies, selection and data collection procedures, and risk-of-bias and GRADE-style certainity assessments for included studies. [file mmc1.pdf]

Appendix

A: PRISMA Checklist

Section and Topic	Item #	Checklist item	Location where item is reported
**TITLE**			
Title	1	Identify the report as a systematic review.	Title page
**ABSTRACT**			
Abstract	2	See the PRISMA 2020 for Abstracts checklist.	Abstract p.1 PROSPERO CRD420251208158.
**INTRODUCTION**			
Rationale	3	Describe the rationale for the review in the context of existing knowledge.	Introduction
Objectives	4	Provide an explicit statement of the objective(s) or question(s) the review addresses.	Key Points & Abstract
**METHODS**			
Eligibility criteria	5	Specify the inclusion and exclusion criteria for the review and how studies were grouped for the syntheses.	Methods
Information sources	6	Specify all databases, registers, websites, organisations, reference lists and other sources searched or consulted to identify studies. Specify the date when each source was last searched or consulted.	& Appendix B
Search strategy	7	Present the full search strategies for all databases, registers and websites, including any filters and limits used.	Appendix B
Selection process	8	Specify the methods used to decide whether a study met the inclusion criteria of the review, including how many reviewers screened each record and each report retrieved, whether they worked independently, and if applicable, details of automation tools used in the process.	Appendix C
Data collection process	9	Specify the methods used to collect data from reports, including how many reviewers collected data from each report, whether they worked independently, any processes for obtaining or confirming data from study investigators, and if applicable, details of automation tools used in the process.	Appendix C
Data items	10a	List and define all outcomes for which data were sought. Specify whether all results that were compatible with each outcome domain in each study were sought (e.g. for all measures, time points, analyses), and if not, the methods used to decide which results to collect.	Methods
	10b	List and define all other variables for which data were sought (e.g. participant and intervention characteristics, funding sources). Describe any assumptions made about any missing or unclear information.	Methods.
Study risk of bias assessment	11	Specify the methods used to assess risk of bias in the included studies, including details of the tool(s) used, how many reviewers assessed each study and whether they worked independently, and if applicable, details of automation tools used in the process.	Appendix D
Effect measures	12	Specify for each outcome the effect measure(s) (e.g. risk ratio, mean difference) used in the synthesis or presentation of results.	Methods
Synthesis methods	13a	Describe the processes used to decide which studies were eligible for each synthesis (e.g. tabulating the study intervention characteristics and comparing against the planned groups for each synthesis (item #5)).	Methods
	13b	Describe any methods required to prepare the data for presentation or synthesis, such as handling of missing summary statistics, or data conversions.	Methods

Section and Topic	Item #	Checklist item	Location where item is reported
	13c	Describe any methods used to tabulate or visually display results of individual studies and syntheses.	Results
	13d	Describe any methods used to synthesize results and provide a rationale for the choice(s). If meta-analysis was performed, describe the model(s), method(s) to identify the presence and extent of statistical heterogeneity, and software package(s) used.	Methods
	13e	Describe any methods used to explore possible causes of heterogeneity among study results (e.g. subgroup analysis, meta-regression).	Discussion
	13f	Describe any sensitivity analyses conducted to assess robustness of the synthesized results.	Methods
Reporting bias assessment	14	Describe any methods used to assess risk of bias due to missing results in a synthesis (arising from reporting biases).	App. D.
Certainty assessment	15	Describe any methods used to assess certainty (or confidence) in the body of evidence for an outcome.	App. D
**RESULTS**			
Study selection	16a	Describe the results of the search and selection process, from the number of records identified in the search to the number of studies included in the review, ideally using a flow diagram.	Results
	16b	Cite studies that might appear to meet the inclusion criteria, but which were excluded, and explain why they were excluded.	Results
Study characteristics	17	Cite each included study and present its characteristics.	Tables 1–2
Risk of bias in studies	18	Present assessments of risk of bias for each included study.	App. D
Results of individual studies	19	For all outcomes, present, for each study: (a) summary statistics for each group (where appropriate) and (b) an effect estimates and its precision (e.g. confidence/credible interval), ideally using structured tables or plots.	Results
Results of syntheses	20a	For each synthesis, briefly summarise the characteristics and risk of bias among contributing studies.	Results
	20b	Present results of all statistical syntheses conducted. If meta-analysis was done, present for each the summary estimate and its precision (e.g. confidence/credible interval) and measures of statistical heterogeneity. If comparing groups, describe the direction of the effect.	Methods
	20c	Present results of all investigations of possible causes of heterogeneity among study results.	Discussion
	20d	Present results of all sensitivity analyses conducted to assess the robustness of the synthesized results.	Methods
Reporting biases	21	Present assessments of risk of bias due to missing results (arising from reporting biases) for each synthesis assessed.	App. D
Certainty of evidence	22	Present assessments of certainty (or confidence) in the body of evidence for each outcome assessed.	App. D
**DISCUSSION**			
Discussion	23a	Provide a general interpretation of the results in the context of other evidence.	Discussion
	23b	Discuss any limitations of the evidence included in the review.	Discussion
	23c	Discuss any limitations of the review processes used.	Discussion
	23d	Discuss implications of the results for practice, policy, and future research.	Discussion
**OTHER INFORMATION**			
Registration	24a	Provide registration information for the review, including register name and registration number, or state that the review	Acknowledgment

Section and Topic	Item #	Checklist item	Location where item is reported
and protocol		was not registered.	
	24b	Indicate where the review protocol can be accessed, or state that a protocol was not prepared.	Acknowledgment
	24c	Describe and explain any amendments to information provided at registration or in the protocol.	Acknowledgment
Support	25	Describe sources of financial or non-financial support for the review, and the role of the funders or sponsors in the review.	Acknowledgment
Competing interests	26	Declare any competing interests of review authors.	Acknowledgment
Availability of data, code and other materials	27	Report which of the following are publicly available and where they can be found: template data collection forms; data extracted from included studies; data used for all analyses; analytic code; any other materials used in the review.	Acknowledgment

B: Information sources:

Search terms:

PubMed (https://pubmed.ncbi.nlm.nih.gov/) :

(("Infant, Newborn"[mh] OR neonate*[tiab] OR newborn*[tiab] OR "Infant, Premature"[mh] OR preterm*[tiab] OR infant*[tiab])) AND (("Infusions, Intraosseous"[mh] OR intraosseous*[tiab] OR "intraosseous access"[tiab] OR "IO access"[tiab])) AND ((success*[tiab] OR efficacy[tiab] OR performance[tiab])) AND english[la] NOT ((animal[mh] NOT human[mh]))

34 results

OVID (https://www.ovid.com/) :

1. exp Infant, Newborn/ OR neonate*.ti,ab. OR newborn*.ti,ab.

2. exp Infant, Premature/ OR preterm*.ti,ab.

3. exp Infusions, Intraosseous/ OR intraosseous*.ti,ab.

4. success*.ti,ab. OR efficacy.ti,ab. OR performance.ti,ab.

5. 1 OR 2

6. 3 AND 4

7. 5 AND 6

8. limit 7 to English language

9. limit 8 to yr="2015 -Current"

10. exp animals/ not humans.sh.

11. 9 not 10

34 results

Cochrane (https://www.cochranelibrary.com/):

#1 (neonate* OR newborn* OR (infant NEXT newborn) OR preterm* OR (premature NEXT infant*) OR infant*)

#2 ((intraosseous NEXT access) OR (intraosseous NEXT infusion) OR (intraosseous NEXT needle) OR intraosseous)

#3 (success OR success* OR efficacy OR effectiveness OR performance)

#4 #1 AND #2 AND #3

#5 (animal*):ti,ab,kw NOT (human*):ti,ab,kw

#6 #4 NOT #5

22 results

C: Selection process:

Two reviewers independently screened titles, abstracts and full texts against pre-specified criteria. Disagreements were resolved by discussion. Cadaveric or anatomical studies were eligible but pre-specified to be analysed separately from live clinical studies.

Data collection process:

To enable a systematic comparison across studies, the key data elements were extracted and are summarized in the following table.

Table 1 Overview of extracted study data

**Category**	**Details**
Article Title	
Date (Publication)	
Source (DOI)	
Author	
Addressed Issues	
Device Description	Actual device: D1<br>Equivalent device: D2<br>Other device: D3

Intended Purpose	Same use: A1<br>Minor deviation: A2<br>Major deviation: A
Risk Management Process	
Population (P)	Appropriate patient group?<br>Applicable: P1<br>Limited: P2<br>Different population: P3
Intervention (I)	
Comparison (C)	
Outcome (O)	
Primary Objective	
Secondary Objective	
Methods	
Results	
Conclusion	
Methodological Philosophy	
Research Method	
Grade of Evidence	High evidence: E1<br>Medium evidence: E2<br>Low evidence: E3
Quality of Information	High quality: R1<br>Medium quality: R2<br>Insufficient quality: R3
Contribution to the Clinical Evaluation	

D: Risk of Bias Assessment

Clinical observational studies were evaluated using the ROBINS-I framework, assessing seven bias domains (confounding, participant selection, intervention classification, deviations, missing data, outcome measurement, and reporting).

Cadaveric and anatomical studies were evaluated using a fit-for-purpose checklist, assessing specimen selection, gestational age ascertainment, bilaterality, imaging confirmation, operator expertise, and predefined success criteria. No reporting bias test and no formal test was performed, so a potential publication bias cannot be excluded."

<u>Appraisal of Observational Clinical Studies (ROBINS-I Framework)</u>

(1) *Pifko et al. (2018): Observational review of paediatric intraosseous needle placement in the paediatric emergency department*

This retrospective, cross-sectional study is assessed as having a moderate risk of bias.

Confounding: The study notes a provider preference for using Manual IO in smaller infants (≤8 kg) and EZ-IO in older children, which makes direct comparison of the devices difficult.

Selection of Participants: The risk of selection bias is low, as the study appears to have included all patients with a documented IO attempt in the paediatric emergency department during the specified period.

Measurement of Outcomes: There is a moderate risk of measurement bias. Data abstraction relied on physician and nursing documentation. The definition of success (documented infusion) is clinically appropriate but dependent on the quality of documentation in the retrospective records.

Missing Data: The retrospective design introduces a moderate risk of bias from missing or incomplete data, which is dependent on the quality of physician and nursing documentation.

(2) *Rijnhout et al. (2024): Effectiveness of intraosseous access during resuscitation: a retrospective cohort study*

This retrospective cohort study is assessed as having a moderate-to-serious risk of bias.

Confounding: There is a moderate-to-serious risk from unmeasured confounders. The study design may have led to an underestimation of the incidence and success rate of IO needle use in the ED due to grammatical inaccuracies in the documentation.

Selection of Participants: The risk of selection bias is low. The study included all patients presenting to the emergency department with documented IO access over a defined period.

Measurement of Outcomes: There is a moderate risk of measurement bias. The success of IO access was based on a retrospective interpretation of file notes, which may introduce subjectivity.

Missing Data: A moderate risk of bias exists due to incomplete data. The study notes that its design may lead to an underestimation of incidence and success rates due to insufficient documentation.

(3) *Mileder et al. (2020): Use of Intraosseous Vascular Access During Neonatal Resuscitation at a Tertiary Center*

This study, using a questionnaire followed by retrospective chart review, is assessed as having a moderate risk of bias.

Confounding: The study is primarily descriptive, but there is a moderate risk of bias in interpreting the findings without full control of confounders. The authors attribute the lower-than expected success rate to the unique anatomy of neonates and the specific device used.

Selection of Participants: There is a moderate risk of selection and recall bias. Patient identification relied on a questionnaire sent to physicians, which is subject to recall error. However, the 100% response rate and subsequent verification via chart review strengthen the methodology and likely mitigate much of this risk.

Measurement of Outcomes: The risk of bias in outcome measurement is low. The primary outcome of successful placement was clearly defined and verified through patient chart reviews, adding a layer of objectivity to the initial survey responses.

Missing Data: There is a moderate risk of bias from missing data, as the study acknowledges the potential for inconsistencies in data reporting inherent in a survey-based methodology.

<u>Cadaveric and Anatomical Studies (Fit-for-Purpose Checklist)</u>

(4) *Harcke et al. (2020): Tibial Intraosseous Insertion in Paediatric Emergency Care: A Review Based upon Post-mortem Computed Tomography*

Specimen Selection: The study used 92 cases from a state medical examiner's office, which was not systematically selected and may therefore introduce bias. The findings are specific to a population of deceased paediatric patients who received emergency care and may not be generalizable to all paediatric patients or survivors.

Age Reporting: Postnatal age was reported appropriately (median age 4 months), which is the relevant metric for this paediatric (non-neonate-specific) population.

Bilaterality: The study design accounted for bilateral insertions, analysing 42 tibial devices across 31 subjects. (11 patients had insertions in both their tibias)

Imaging Confirmation: This is a major strength. Post-mortem computed tomography (PMCT) was used to precisely determine the final needle tip position, providing an objective and accurate assessment of placement success.

Operator Expertise: This is a significant limitation. The study could not track the experience level or professional category of the personnel performing the IO insertions in the clinical setting, which is a critical variable influencing success rates.

(5) *Sengasai et al. (2024): A prospective evaluation of tibial insertion sites for intraosseous needles in Asian neonates*

Specimen Selection: The study used a prospective design with clear inclusion and exclusion criteria for neonatal cadavers, representing a systematic approach to specimen selection.

Gestational Age Reporting: Gestational age and birth weight were reported, and data were stratified by birth weight category, which is a robust approach for this population.

Bilaterality: The methodology accounted for bilaterality by alternating insertions between the left and right legs.

Imaging Confirmation: This is a key strength. Placement success was confirmed using CT scans with contrast media to visualize distribution within the marrow cavity, providing definitive evidence of functional placement.

Operator Expertise: This is a potential limitation. All insertions were performed by a single principal investigator. While this ensures consistency, it may inflate the success rate compared to real-world scenarios involving operators with varying levels of experience.

(6) *Fuchs et al. (2018): Anatomical investigations on intraosseous access in stillborn*

Specimen Selection: The study used a small convenience sample of 16 formaldehyde-fixed stillborn. The use of fixed tissues is a significant limitation, as formalin alters the physical properties of bone and soft tissue, which may not accurately reflect conditions in living neonates.

Bilaterality: The investigation involved both legs of the cadavers, addressing bilaterality.

Imaging Confirmation: A major strength of the study was the use of spectral-CT to confirm needle placement, providing high-quality, objective data on insertion accuracy.

Operator Expertise: Insertions were performed by a single experienced investigator. As with the Sengasai et al. study, this ensures procedural consistency but limits the generalizability of the success rates to a wider range of clinical providers.

GRADE-Style certainty:

<u>Observational Clinical Studies</u>

(1) *Pifko et al. (2018): Observational review of paediatric intraosseous needle placement in the paediatric emergency department*

Small-Study Effects and Reporting Bias:

This study primary limitation is its small sample size (50 patients over 8.5 years), which provides inadequate statistical power for definitive conclusions. The infrequency of the procedure in the studied emergency department suggests a lack of routine practice, which may confound the results. A risk of reporting bias exists due to the retrospective design, which relies on the quality of physician and nursing documentation. Furthermore, a provider preference for using Manual IO in infants and EZ-IO in older children introduces a potential selection bias that affects the direct comparison of the devices.

GRADE-Style Certainty Statement:

The clinical evidence regarding the comparison of Manual IO and EZ-IO devices in small infants (≤8 kg) from this single-centre study is of Low to Very Low Certainty.

(2) *Rijnhout et al. (2024): Effectiveness of intraosseous access during resuscitation: a retrospective cohort study*

Small-Study Effects and Reporting Bias:

This retrospective database study may suffer from reporting bias, as the authors note that insufficient documentation in the emergent setting could lead to an underestimation of IO use and success rates. A key limitation is the inability to analyse critical confounding variables like operator experience or needle length, as this data was not consistently recorded.

GRADE-Style Certainty Statement:

The clinical neonatal evidence from this study, specifically for infants under six months, is of Low to Very Low Certainty. This is based on the authors; own conclusion that caution is warranted for this age group due to their markedly lower success rates and the study's retrospective nature with significant missing data on key variables.

(3) *Mileder et al. (2020): Use of Intraosseous Vascular Access During Neonatal Resuscitation at a Tertiary Center*

Small-Study Effects and Reporting Bias: This study is highly at risk to small-study effects, having identified only 12 neonates undergoing 15 IO attempts over a 61-month period. This extremely low frequency (3 attempts per year) limits the generalizability of its findings.

GRADE-Style Certainty Statement: The clinical neonatal evidence from this single tertiary canter is of Very Low Certainty. This rating is due to the extremely small sample size, the rarity of the event in this setting, and the methodological limitations of a survey-informed retrospective review.

<u>Cadaveric/Anatomical Studies</u>

(4) *Harcke et al. (2020): Tibial Intraosseous Insertion in Paediatric Emergency Care: A Review Based upon Post-mortem Computed Tomography*

Small-Study Effects and Certainty Limitations:

This study, a post-mortem review of IO insertions performed during clinical care, is limited by its sample size; the authors noted that the small number of patients precluded finding statistically significant differences based on needle length alone. The primary limitation affecting certainty is the inability to determine crucial variables from the clinical event, such as the operator experience. The study measured only anatomical placement, not functional infusion success.

GRADE-Style Certainty Statement:

The certainty of translating these findings to clinical neonatal success is Low. The evidence is derived from a post-mortem review of deceased children, which cannot account for live tissue response.

(5) *Sengasai et al. (2024): A prospective evaluation of tibial insertion sites for intraosseous needles in Asian neonates*

Small-Study Effects and Certainty Limitations:

While methodologically robust for a cadaveric study, a form of small-study effect was observed in the subgroup analysis. The overall success rate of 86.8% dropped significantly to 66.7% in very-low birthweight (VLBW) infants, indicating that findings from the broader cohort do not reliably translate to the smallest and most vulnerable subjects. Certainty is also limited by the use of a single, highly trained operator, which may not reflect typical clinical practice and could inflate success rates.

GRADE-Style Certainty Statement:

The certainty of translating these anatomical success rates directly to clinical success is Low. Although the study provides high-certainty anatomical guidance, the use of a cadaveric model (which lacks physiological responses).

(6) *O*

Small-Study Effects and Certainty Limitations:

The authors explicitly state that their results are limited by the small sample size (16 stillborn). This small sample size likely contributed to the model's failure to achieve statistical significance despite a large observed effect. The greatest limitation to certainty is the use of formaldehyde-fixed stillborn, as the fixation process alters tissue composition and may not accurately represent the mechanics of insertion in a living neonate.

GRADE-Style Certainty Statement:

The certainty of translating these findings to clinical success in live neonatal resuscitation is Very Low. This is due to the fundamental limitations of using a small sample of formaldehyde-fixed stillborn cadavers, which differs significantly from the dynamic physiological environment of a live patient.
